# Humoral immune response to circulating SARS-CoV-2 variants elicited by inactivated and RBD-subunit vaccines

**DOI:** 10.1038/s41422-021-00514-9

**Published:** 2021-05-21

**Authors:** Yunlong Cao, Ayijiang Yisimayi, Yali Bai, Weijin Huang, Xiaofeng Li, Zhiying Zhang, Tianjiao Yuan, Ran An, Jing Wang, Tianhe Xiao, Shuo Du, Wenping Ma, Liyang Song, Yongzheng Li, Xiang Li, Weiliang Song, Jiajing Wu, Shuo Liu, Xuemei Li, Yonghong Zhang, Bin Su, Xianghua Guo, Yangyang Wei, Chuanping Gao, Nana Zhang, Yifei Zhang, Yang Dou, Xiaoyu Xu, Rui Shi, Bai Lu, Ronghua Jin, Yingmin Ma, Chengfeng Qin, Youchun Wang, Yingmei Feng, Junyu Xiao, Xiaoliang Sunney Xie

**Affiliations:** 1 Beijing Advanced Innovation Center for Genomics (ICG), Peking University, Beijing, China; 2 Biomedical Pioneering Innovation Center (BIOPIC), Peking University, Beijing, China; 3 School of Life Sciences, Peking University, Beijing, China; 4 Joint Graduate Program of Peking-Tsinghua-NIBS, School of Life Sciences, Tsinghua University, Beijing, China; 5 Division of HIV/AIDS and Sex-transmitted Virus Vaccines, Institute for Biological Product Control, National Institutes for Food and Drug Control (NIFDC) and WHO Collaborating Center for Standardization and Evaluation of Biologicals, Beijing, China; 6 State Key Laboratory of Pathogen and Biosecurity, Beijing Institute of Microbiology and Epidemiology, Academy of Military Medical Sciences, Beijing, China; 7 Peking-Tsinghua Center for Life Sciences, Academy for Advanced Interdisciplinary Studies, Peking University, Beijing, China; 8 Beijing Youan Hospital, Capital Medical University, Beijing, China; 9 School of Pharmaceutical Sciences, IDG/McGovern Institute for Brain Research, Tsinghua University, Beijing, China; 10 Vazyme Biotech Co., Ltd, Nanjing, Jiangsu China; 11 CAS Key Laboratory of Microbial Physiological and Metabolic Engineering, Institute of Microbiology, Chinese Academy of Sciences, Beijing, China; 12 Beijing Ditan Hospital, Capital Medical University, Beijing, China

**Keywords:** Immunology, Cryoelectron microscopy, X-ray crystallography

## Abstract

SARS-CoV-2 variants could induce immune escape by mutations on the receptor-binding domain (RBD) and N-terminal domain (NTD). Here we report the humoral immune response to circulating SARS-CoV-2 variants, such as 501Y.V2 (B.1.351), of the plasma and neutralizing antibodies (NAbs) elicited by CoronaVac (inactivated vaccine), ZF2001 (RBD-subunit vaccine) and natural infection. Among 86 potent NAbs identified by high-throughput single-cell VDJ sequencing of peripheral blood mononuclear cells from vaccinees and convalescents, near half anti-RBD NAbs showed major neutralization reductions against the K417N/E484K/N501Y mutation combination, with E484K being the dominant cause. VH3-53/VH3-66 recurrent antibodies respond differently to RBD variants, and K417N compromises the majority of neutralizing activity through reduced polar contacts with complementarity determining regions. In contrast, the 242–244 deletion (242–244Δ) would abolish most neutralization activity of anti-NTD NAbs by interrupting the conformation of NTD antigenic supersite, indicating a much less diversity of anti-NTD NAbs than anti-RBD NAbs. Plasma of convalescents and CoronaVac vaccinees displayed comparable neutralization reductions against pseudo- and authentic 501Y.V2 variants, mainly caused by E484K/N501Y and 242–244Δ, with the effects being additive. Importantly, RBD-subunit vaccinees exhibit markedly higher tolerance to 501Y.V2 than convalescents, since the elicited anti-RBD NAbs display a high diversity and are unaffected by NTD mutations. Moreover, an extended gap between the third and second doses of ZF2001 leads to better neutralizing activity and tolerance to 501Y.V2 than the standard three-dose administration. Together, these results suggest that the deployment of RBD-vaccines, through a third-dose boost, may be ideal for combating SARS-CoV-2 variants when necessary, especially for those carrying mutations that disrupt the NTD supersite.

## Introduction

Recently, several new SARS-CoV-2 variants emerged worldwide, raising concerns that their recognition by the human immune system may be affected. B.1.1.7, also known as 501Y.V1, was discovered in the United Kingdom in September 2020,^1^ and it subsequently increased in prevalence and spread to other countries and continents. 501Y.V1 is associated with a set of mutations in its spike (S) protein, including ΔH69/V70 and ΔY144 in N-terminal domain (NTD), N501Y in receptor-binding domain (RBD), and P681H near the furin cleavage site. B.1.351, also known as 501Y.V2, initially emerged in late 2020 in South Africa and quickly became a dominant strain in the Eastern Cape.^2^ 501Y.V2 is associated with multiple S mutations, which could be divided into two main subsets, one is clustered in NTD (L18F, D80A, D215G, 242–244Δ, and R246I), and the other is clustered in RBD (K417N, E484K, and N501Y). P.1, also known as 501Y.V3, is discovered and announced in January 2021 in Brazil.^3^ 501Y.V3 shares multiple common mutations in the RBD region with 501Y.V2, including N501Y, E484K, and K417N, but lacks 242–244Δ and R246I in the NTD. Multiple reports have been released to claim that these variants, especially 501Y.V2, could reduce the neutralization capability of convalescent plasma and therapeutic NAbs.^4,5^ Importantly, Oxford-AstraZeneca and Novavax vaccines have shown reduced efficacy in clinical trials conducted in South Africa.^6,7^ Indeed, vaccines using the full-length S as the antigen, including spike-mRNA and spike-subunit vaccines, have also shown a decline in neutralization activity toward 501Y.V2.^8,9^ However, how these mutations affect vaccines utilizing different antigen constructs, such as RBD or inactivated whole viruses, is unclear and requires an immediate survey.

Therefore, we recruited 30 volunteers who received either CoronaVac or ZF2001 to examine the resistance to 501Y.V2 mutants 2 weeks after the final dose (Supplementary information, Table S1). CoronaVac is a SARS-CoV-2 inactivated vaccine that has already been authorized for emergency use in China, Brazil, and multiple other countries.^10^ Ten CoronaVac vaccinee volunteers (designated as I1–I10) were recruited at Youan Hospital after a standard 0/21 two-dose vaccination. ZF2001 is an RBD-dimer subunit vaccine that has been authorized to be deployed in China and Uzbekistan.^11,12^ Twenty ZF2001 vaccinee volunteers (designated as R1–R20) were also recruited after a 0/30/60 three-dose vaccination (*n* = 10) or a 0/30/140 extended third-dose vaccination (*n* = 10). None of the volunteers was prior infected by SARS-CoV-2. Besides, 10 convalescent samples (designated as CE1–CE10) collected 1.3 months after infection are also included in the study to compare the variances in 501Y.V2 tolerance of vaccinees (Supplementary information, Table S1).

## Results

### RBD and NTD mutations carried by 501Y.V2 abolish a large proportion of neutralizing activity of NAbs elicited in convalescents and vaccinees

**Fig. 1:**
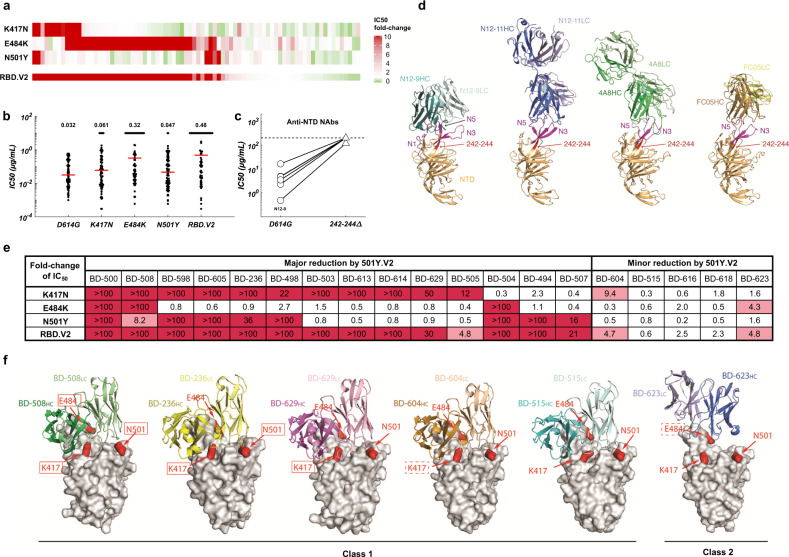
Responses of anti-RBD and anti-NTD SARS-CoV-2 NAbs to 501Y.V2. **a** IC_50_ fold-changes of 80 potent anti-RBD NAbs against pseudovirus carrying 501Y.V2 RBD mutations. **b** The geometric means of IC_50_ values of 80 anti-RBD NAbs against the indicated mutants. For NAbs with IC_50_ > 10 μg/mL, IC_50_ is designated as 10 μg/mL. **c** The IC_50_ values of six potent anti-NTD NAbs against pseudovirus carrying D614G or 242–244Δ. **d** Structure analyses of the NTD NAbs. The N1, N3, and N5 loops of NTD are highlighted in magenta. Residues 242–244 are highlighted in red. **e** IC_50_ fold-changes of VH3-53/VH3-66 NAbs against pseudovirus carrying 501Y.V2 RBD mutations. Red indicates major fold-change larger than 10-fold. Pink indicates minor fold-change between 3- and 10-fold. **f** Structure analyses of the interaction between VH3-53/VH3-66 NAbs and RBD. RBD is shown as a surface view, whereas the NAbs are shown as ribbons. Red solid rectangles indicate that the mutation would result in a major decrease in antibody binding. Red dashed rectangles indicate that the mutation would result in a minor decrease in antibody binding.

To understand how 501Y.V2 mutations affect SARS-CoV-2 neutralization, we first isolated RBD- and NTD-specific memory B cells by fluorescence-activated cell sorting (FACS) from peripheral blood mononuclear cells (PBMCs) of CoronaVac and ZF2001 vaccinees, as well as COVID-19 convalescents (Supplementary information, Fig. S1). High-throughput single-cell VDJ sequencing was applied to obtain paired heavy- and light-chain VDJ sequences to generate antibody sequences.^13,14^ Combined with the antibodies that we previously identified from convalescents,^13^ a total of 831 anti-Spike antibodies were identified from enriched antigen-specific memory B cell clonotypes, and 86 potent SARS-CoV-2 NAbs were selected to test their neutralization ability against circulating mutants, including 501Y.V2 (Supplementary information, Table S2). SARS-CoV-2 VSV-pseudovirus carrying 501Y.V2 mutations were constructed and used to survey how a large collection of NAbs react to 501Y.V2 variants (Supplementary information, Fig. S2).^5^ Strikingly, 50 out of 80 anti-RBD NAbs showed a > 3-fold reduction of neutralization toward pseudovirus carrying K417N/E484K/N501Y mutations (RBD.V2), among which 43 anti-RBD NAbs display a > 10-fold increase in half-maximal inhibitory concentration (IC_50_) (Fig. 1a). The majority of neutralization reduction was caused by E484K, which could completely abolish antibodies’ neutralization activity, resulting in a > 10-fold increase on average in IC_50_ values (Fig. 1b). N501Y and K417N mutations also contributed to neutralization reduction, but to a much lower degree than E484K, resulting in a 1.5-fold and a 1.9-fold increase in IC_50_, respectively. On the other hand, all 6 anti-NTD NAbs have lost the neutralization ability against 501Y.V2 carrying 242–244Δ (Fig. 1c), suggesting that the epitopes inducing neutralization on the NTD are much less diverse than those on the RBD, which is consistent with previous observation of NTD supersite.^15^ To reveal the molecular mechanism of these NAbs, we characterized their specific interactions with NTD. We determined the cryo-EM structure of N12-9 in complex with the S trimer together with two RBD-specific NAbs BD-368-2 and BD-604 (Fig. 1d; Supplementary information, Fig. S3). We also determined the crystal structure of N12-11 bound to separate NTD (Fig. 1d). N12-9 interacts with three NTD surface loops, N1, N3 and N5, whereas N12-11 only binds to N3 and N5. The surface region formed by these three loops appears to be the focus of many NTD NAbs, such as 4A8 and FC05,^16,17^ which is defined as an NTD antigenic supersite.^15,18^ Importantly, residues 242–244 belong to part of a strand that precedes the N5 loop, and therefore deletion of these residues would drastically alter the conformation of N5. Since N5 is involved in interacting with all the NTD NAbs characterized to date, the 501Y.V2 would inevitably evade most NTD-elicited antibody responses.

### VH3-53/VH3-66 recurrent NAbs react diversely toward 501Y.V2 mutants despite structural similarities

To further investigate the diversity of anti-RBD NAbs against 501Y.V2 variants, 18 additional VH3-53/VH3-66 recurrent NAbs previously isolated based on VDJ combinations were tested for 501Y.V2 tolerance (Supplementary information, Table S2).^19^ Although these commonly found recurrent NAbs share similar structural interactions with RBD,^20,21^ their reactivity to 501Y.V2 is not uniform (Fig. 1e). The neutralization ability of most VH3-53/VH3-66 recurrent NAbs was abolished by RBD.V2; interestingly, there do exist NAbs that are not affected. To assess the origin of diversity, we performed structural analyses (Fig. 1f). BD-508, BD-236, BD-629, BD-604, and BD-515 are class 1 NAbs and displayed a similar RBD-binding pose.^22^ In the crystal structure of RBD in complex with the Fab of BD-508, Lys417 and Glu484 form hydrogen bonds with Gln100 and Tyr102 in heavy chain complementarity determining region 3 (CDR_H_3), respectively, whereas Asn501 interacts with Gln27 and Ser28 in light chain complementarity determining region 1 (CDR_L_1). Thus, K417N, or E484K, or N501Y found in the 501Y.V2 variant would all greatly reduce the interaction between RBD and BD-508. In the structure of RBD/BD-236, Lys417 forms a salt bridge with Glu101 in CDR_H_3, and Asn501 is buried by Ile29 and Ser30 in CDR_L_1. Glu484 does not directly contact BD-236. As a result, only mutation of Lys417 or Asn501 decreased the neutralizing activity of BD-236. BD-629 is susceptible to the change of Lys417, since Lys417 interacts with Gly100 and Asp101 in CDR_H_3.^19^ BD-604 is also slightly affected by the K417N mutation.^19^ In contrast to these NAbs, none of these RBD residues are involved in interacting with BD-515, another class 1 VH3-53/VH3-66 NAb; therefore, BD-515 is completely resistant to their mutations. BD-623 has a long CDR_H_3 and belongs to the class 2 VH3-53/VH3-66 NAbs.^22,23^ In the structure of RBD/BD-623, Lys417 and Asn501 do not contact BD-623. The main chain carbonyl group of Glu484 forms a hydrogen bond with Tyr58 in CDR_H_2. As this interaction is mediated by the main chain group, the E484K substitution only has a limited impact on the neutralizing power of BD-623. Collectively, structural analyses demonstrated the accuracy of pseudovirus assays and showed diverse interactions between anti-RBD NAbs and RBD. Together, these results suggest that anti-RBD NAbs are more beneficial against 501Y.V2 variants than anti-NTD NAbs, due to the high diversity that anti-RBD NAbs exhibit in contrast to anti-NTD NAbs.^15^

### The effects of circulating mutants on convalescents and CoronaVac vaccinees

**Fig. 2:**
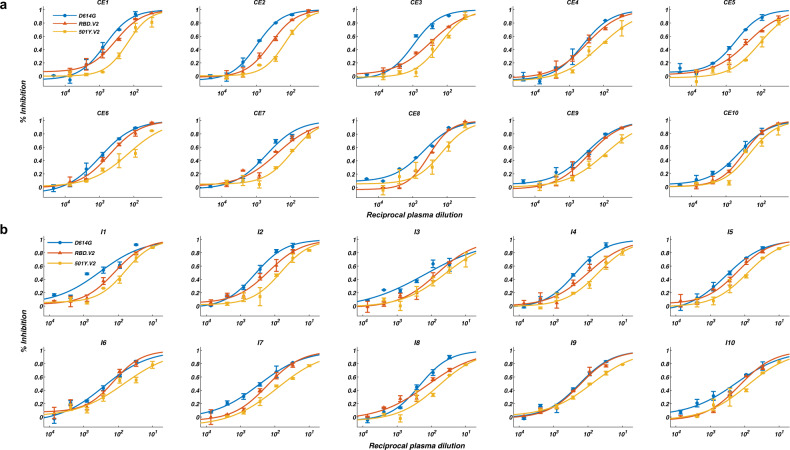
Pseudovirus neutralization assays of convalescent plasma and Coronavac vaccinee plasma against 501Y.V2 mutants. **a** VSV-pseudovirus neutralization assays measuring neutralizing ability of convalescent plasma against D614G (blue), RBD.V2 (red), and 501Y.V2 (yellow) mutants. **b** VSV-pseudovirus neutralization assays measuring neutralizing ability of CoronaVac vaccinee plasma against D614G (blue), RBD.V2 (red), and 501Y.V2 (yellow) mutants. All experiments were reproduced at least twice.

**Fig. 3:**
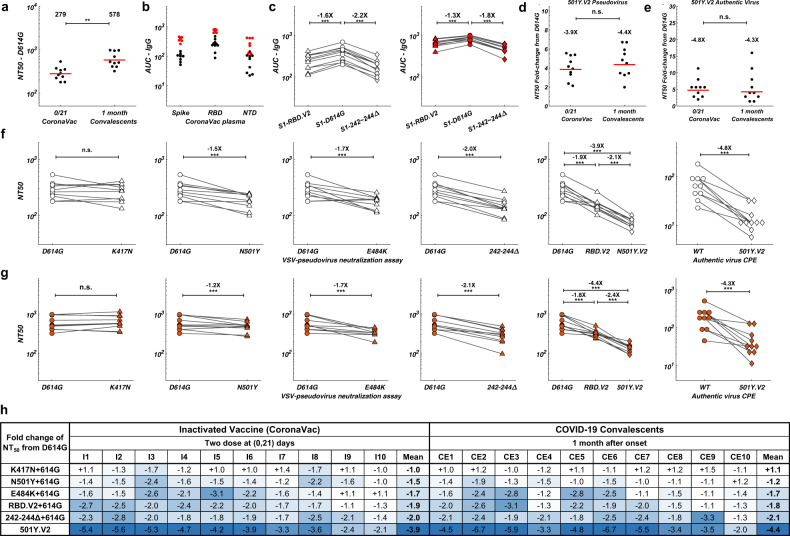
Binding and neutralization of 501Y.V2 by convalescent and CoronaVac vaccinee plasma. **a** NT_50_ values for COVID-19 convalescent plasma (CE) and CoronaVac vaccinee plasma (I) using D614G mutant pseudovirus neutralization assay. Statistical significance was determined using one-tailed *t*-test (**P < 0.01). **b** Plasma anti-IgG ELISA reactivity to S(ECD), RBD, and NTD. Normalized area under the curve (AUC) values are plotted. Black dots stand for CoronaVac vaccinees, and red dots stand for convalescents. **c** Comparison of anti-IgG ELISA AUC values among RBD.V2 (triangle), D614G (circle), and 242–244Δ (diamond) mutants of S1. White stands for CoronaVac vaccinees, and red stands for convalescents. Statistical significance was determined using paired one-tailed *t*-test (***P < 0.001). **d** NT_50_ fold-change from D614G to 501Y.V2 for convalescent and CoronaVac vaccinee plasma by pseudovirus neutralization assay. Statistical significance was determined using one-tailed *t*-test (n.s., no significance). **e** NT_50_ fold-change from WT to 501Y.V2 for convalescent and CoronaVac vaccinee plasma by authentic virus neutralization assay. Statistical significance was determined using one-tailed *t*-test (n.s., no significance). **f** Comparison of NT_50_ values by pseudovirus or authentic virus neutralization assay between WT/D614G and the indicated mutants for CoronaVac vaccinee plasma samples. **g** Comparison of NT_50_ values by pseudovirus or authentic virus neutralization assay between WT/D614G and the indicated mutants for CE samples. **h** Summary of the fold change of NT_50_ for the indicated mutants from D614G. Color gradient indicates fold change values ranging from +1 (white) to −6.7 (blue). All plotted values and horizontal bars in this figure indicate the geometric mean.

Next, plasma samples of convalescents and CoronaVac vaccinees were tested against 501Y.V2 variants to confirm whether plasma reacts similarly to the NAbs elicited (Supplementary information, Table S3). Plasma’s efficacy against SARS-CoV-2 is first validated by VSV-pseudovirus (D614G) neutralization assay (Fig. 2a, b). All 20 plasma samples showed neutralization activity against SARS-CoV-2, while CoronaVac vaccinee plasma samples displayed a lower half-maximal neutralizing titer (NT_50_) compared to CE (Fig. 3a). Anti-IgG ELISA measurement showed that both anti-RBD and anti-NTD antibodies were elicited in convalescent and CoronaVac vaccinee plasma (Fig. 3b). Purified S1 proteins with RBD and NTD mutations carried by 501Y.V2 were used to examine the loss of antibody binding of plasma to variants (Fig. 3c). Convalescent and CoronaVac vaccinee plasma samples showed a clear reduction in IgG reactivity to both RBD- and NTD-mutated S1 proteins. VSV-pseudovirus neutralization assays showed that all plasma samples displayed an NT_50_ reduction against 501Y.V2 (Fig. 3d). Similar to NAb results, E484K largely contributed to the loss of plasma neutralizing activity caused by RBD mutations (Fig. 3f, g). N501Y also accounted for NT_50_ reduction but less than E484K, while K417N did not reduce neutralization by plasma. For NTD mutations, 242–244Δ cause 2–3-fold reduction in NT_50_ titers (Fig. 3f, g). The reductions caused by RBD and NTD mutations are additive, creating a total of near 4-fold decrease in NT_50_ against 501Y.V2 pseudovirus (Fig. 3h). Importantly, authentic virus cytopathic effect (CPE) assays using wild type (WT) and 501Y.V2 variants showed comparable NT_50_ reduction in pseudovirus assay (Fig. 3e). Together, 501Y.V2 would cause a major reduction in neutralization by convalescent plasma and CoronaVac vaccinee plasma through E484K and 242–244Δ, with the effects being additive.

### The effects of circulating mutants on RBD-subunit vaccine

**Fig. 4:**
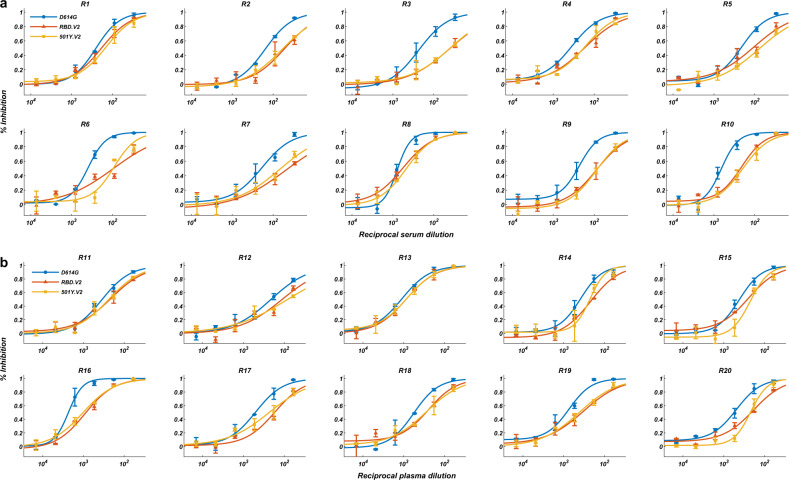
Pseudovirus neutralization assays of ZF2001 vaccinee plasma against 501Y.V2 mutants. Pseudovirus neutralization assays measuring plasma neutralizing ability against D614G (blue), RBD.V2 (red), and 501Y.V2 (yellow) mutants. **a** R1–R10 stand for serum samples collected from ZF2001 vaccinees who received Day 0, 30, 60 administration. **b** R11–R20 stand for plasma samples collected from ZF2001 vaccinees who received Day 0, 30, 140 administration. All experiments were reproduced at least twice.

**Fig. 5:**
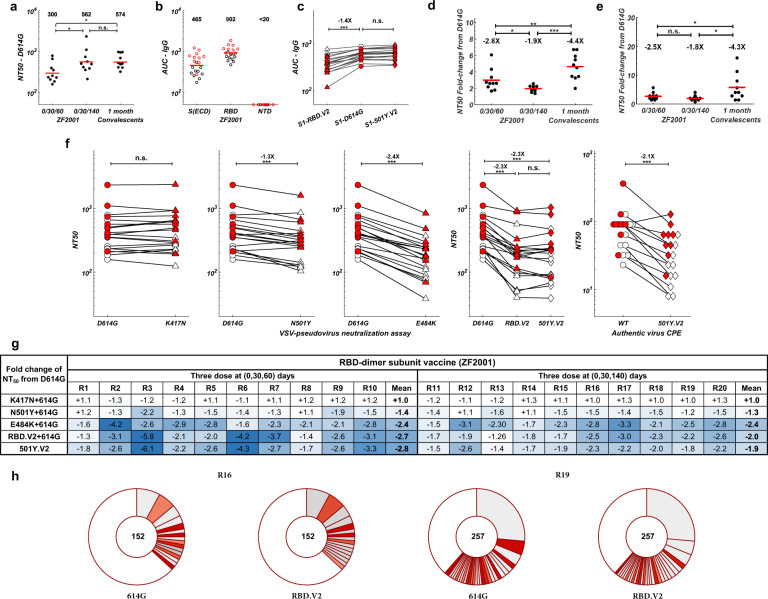
Binding and neutralization of 501Y.V2 by ZF2001 vaccinee plasma. **a** NT_50_ values for ZF2001 vaccinee plasma/sera using D614G mutant pseudovirus neutralization assay. Statistical significance was determined using one-tailed *t*-test (*P < 0.05; n.s., no significance). **b** Plasma/serum anti-IgG ELISA reactivity to S(ECD), RBD, and NTD. Normalized AUC values are plotted. Black circles stand for R1–R10, and red circles stand for R11–R20. **c** Comparison of anti-IgG ELISA AUC values among RBD.V2 (triangle), D614G (circle), and 242–244Δ (diamond) mutants of S1. White stands for R1–R10, and red stands for R11–R20. Statistical significance was determined using paired one-tailed *t*-test (***P < 0.001; n.s., no significance). **d** NT_50_ fold-change from D614G to 501Y.V2 for ZF2001 vaccinee plasma/sera by pseudovirus neutralization assay. Statistical significance was determined using one-tailed *t*-test (*P < 0.05; **P < 0.01; ***P < 0.001). **e** NT_50_ fold-change from WT to 501Y.V2 for ZF2001 vaccinee plasma/sera by authentic virus neutralization assay. Statistical significance was determined using one-tailed *t*-test (*P < 0.05; n.s., no significance). **f** Comparison of NT_50_ values by pseudovirus or authentic virus neutralization assay between WT/D614G and the indicated mutants for ZF2001 vaccinee plasma samples. **g** Summary of the fold change of NT_50_ for the indicated mutants from D614G. Color gradient indicates fold change values ranging from +1 (white) to −6.1 (blue). All plotted values and horizontal bars in this figure indicate the geometric mean. **h** Pie charts indicating the distribution of antibody sequences from two ZF2001 vaccinees. The number in the inner circle indicates the number of single cells analyzed. Slice size is proportional to the number of clonal enrichment frequencies. The color of the slice indicates neutralization potency toward pseudovirus mutants. Gray, expressed antibodies but with low or no neutralization (IC_50 _> 1 μg/mL) and darker gray indicates lower potency; red, high neutralization potency (IC_50 _< 1 μg/mL) and darker red indicates higher potency; white, not expressed.

As for the RBD-dimer subunit vaccine ZF2001, all 20 volunteers exhibited neutralization activities against SARS-CoV-2 (Fig. 4a, b). The extended three-dose group’s plasma showed significantly higher NT_50_ than that of the standard 0/30/60 administration group (Fig. 5a). As expected, ZF2001 did not induce anti-NTD antibodies (Fig. 5b); thus, IgG binding is only affected by 501Y.V2 RBD mutants but not NTD mutants (Fig. 5c). Importantly, this property causes ZF2001 to be only compromised by RBD mutations, with the 242–244Δ showing no effects (Fig. 5f). Furthermore, we in vitro expressed all clonally-enriched antibody sequences from two ZF2001 vaccinees (Fig. 5h), and tested the antibodies against D614G and RBD.V2 pseudovirus. The results demonstrated that individuals are capable of inducing highly diverse anti-RBD NAbs that react differently toward 501Y.V2. Together, these enable ZF2001 vaccinees to exhibit a two times higher tolerance to 501Y.V2 variants in both pseudovirus and authentic virus assays than CoronaVac vaccinees and convalescents (Fig. 5d, e). The high tolerance and neutralization efficacy toward 501Y.V2 make RBD-vaccines ideal to counter mutants that bear potent NTD mutations, such as 242–244Δ. However, how RBD-vaccine efficacy and mutation tolerance change with time still needs to be investigated. Interestingly, the extended three-dose ZF2001 group showed less NT_50_ reduction than the standard three-dose group (Fig. 5g). This phenomenon is likely due to additional antibody maturations acquired through continuous hypermutations before the third-dose boost, and needs to be confirmed in longitudinal studies. A similar observation is reported in a longitudinal study on COVID-19 convalescents between 1.3 months and 6.2 months after infection, where the NAbs from convalescents 6.2 months post infection display higher somatic mutations and better tolerance to mutants.^24^ Nevertheless, the fact that an extended three-dose (0/30/140) could still stimulate strong neutralization activity makes ZF2001 a suitable third-dose boost to counter 501Y.V2 when necessary.

## Discussion

NAbs targeting both NTD and RBD are present in the convalescent plasma and can be elicited by the inactivated SARS-CoV-2 vaccine. The NTD-targeting NAbs aim at a common region on NTD and can be easily escaped by the SARS-CoV-2 variants containing the 242–244Δ mutation, such as 501Y.V2, since deletion of these residues would disrupt the structural integrity of this antigenic supersite. In contrast, the RBD-targeting NAbs recognize diverse areas on RBD. For example, the VH3-53/VH3-66 recurrent NAbs can display two types of binding to RBD: the majority of them contain short CDR_H_3s and can only bind to RBDs that adopt the “up” conformation, whereas a subset of them feature longer CDR_H_3s and can interact with both “up” and “down” RBDs. Due to difference in their binding epitopes, these NAbs exhibit different degrees of sensitivity to 501Y.V2. As we described above, some of the NAbs are extremely vulnerable to the mutations of Lys417, Glu484, and Asn501, whereas others are more tolerant. With the continuous spreading of SARS-CoV-2, it is likely that more RBD mutations would emerge, and some of the mutations might reduce neutralization by altering RBD structure instead of simply perturbing the NAb binding surface. For example, a yeast screening experiment showed that the E406W mutation on RBD abolishes neutralization by both NAbs in the REGN-COV2 cocktail, although this residue does not directly interact with either of them.^25^ On the other hand, the same mutation only slightly decreases neutralization by LY-CoV016, another anti-RBD NAb. From a vaccine point of view, it is likely more beneficial to use an immunogen that can induce and enrich RBD-directed NAbs, which are more resistant to SARS-CoV-2 mutations due to their diverse modes of RBD binding, and can therefore protect against a broader spectrum of viral variants.

Recently, two articles^9,26^ reported the resistance of circulating variants to Pfizer and Moderna mRNA vaccines, including B.1.351 and B.1.1.7. B.1.351/501Y.V2 would also cause a high reduction in neutralization titers of mRNA vaccinee’s plasma, which is consistent with our results. However, due to the different experimental settings of the pseudovirus neutralization assays, it is difficult to directly compare the neutralization fold-changes among inactivated vaccines, RBD recombinant subunit vaccines, and mRNA vaccines toward 501Y.V2. Nevertheless, the trend is the same; that is, E484K and 242–244Δ would greatly influence vaccine efficacy.

Due to the concerns brought by the new variants, SARS-CoV-2 vaccines utilizing 501Y.V2 strain as the antigen are being produced and are waiting to be evaluated. Preliminarily, our collaborators and we found that mRNA vaccine using 501Y.V2 strain does not seem as effective as that using the WT strain in mice, in terms of neutralization activity in sera;^27^ in addition, 501Y.V2-vaccinated mice tend to show a reduced neutralization efficacy toward WT SARS-CoV-2 compared to 501Y.V2 variants. These results suggest that the new variant vaccine might not be suitable to be deployed as a stand-alone vaccine but should be combined with the current vaccines to construct bivalent vaccines. Fortunately, the new variant strains do not spread as rapidly as the D614G strain did, such that the rapid deployment of vaccines using WT antigens might be sufficient to stop the pandemic. However, once variant strains spread on a large scale and in a rapid way, especially those mutants that carry mutations disrupting the NTD supersite, a third booster shot of vaccines utilizing RBD as the antigen should be ideal for providing adequate protection.

## Materials and methods

### Serum separation

Whole blood obtained from volunteers were left undisturbed to clot at room temperature for 2 h. The clot was removed by centrifuging at 1000 × *g* for 5 min in a refrigerated centrifuge. Then the liquid component serum was apportioned and transported on dry ice.

### Plasma, PBMC and B cell collection

Whole blood samples were subjected to Ficoll (Cytiva, 17-1440-03) gradient centrifugation after 1:1 dilution in PBS (Invitrogen, C10010500BT) + 2% FBS (Gibco, A3160901). After centrifugation, plasma was collected from upper layer and cells were harvested at the interface. PBMCs were further prepared through centrifugation, red blood cell lysis (InvitrogenTM eBioscience^TM^ 1X RBC Lysis Buffer, 00-4333-57) and washing steps. Some samples were stored in FBS with 10% DMSO (Sigma-Aldrich, D4540) in liquid nitrogen if not used for downstream process immediately. All PBMC samples were shipped on dry ice. Cryopreserved PBMCs were thawed in DPBS + 2% FBS (STEMCELL, 07905). On the day of sorting, B cells were enriched from fresh or previously frozen PBMCs by immunomagnetic negative selection using the EasySep™ Human B Cell Enrichment Kit (STEMCELL, 17954). Non-B cells are labeled with magnetic beads and separated using an EasySep™ magnet. Purified B cells were eluted and washed in PBS containing 2% (v/v) FBS and 1 mM EDTA. Purified B cells were counted by using 0.4% (w/v) trypan blue stain (Invitrogen, T10282) and Countess Automated Cell Counter according to the manufacturer’s protocol (Invitrogen).

### Antigen protein synthesis

Biotinylated SARS-CoV-2 Spike RBD-AVI & His Recombinant Protein (Sino Biological Inc., 40592-V27H-B), Biotinylated NTD-His & AVI Recombinant Protein (Sino Biological Inc., 40591-V49H-B), Biotinylated Nucleocapsid-AVI & His recombinant Protein (Sino Biological Inc., 40588-V27B-B), Biotinylated Spike S2 ECD-His Recombinant Protein (Sino Biological Inc., 40590-V08B-B), Biotinylated Spike S1-AVI & His Recombinant Protein (Sino Biological Inc., 40591-V27H-B) were used in this study. DNA sequences encoding the SARS-CoV-2 Spike RBD-AVI & His Recombinant Protein (YP_009724390.1) (Arg319–Phe541), Spike S1 NTD (YP_009724390.1) (Met1–Ser305), Nucleocapsid Protein (YP_009724397.2 (335Gly/Ala)) (Met1–Ala419), Spike Protein (S2 ECD) (YP_009724390.1) (Ser686–Pro1213), and Spike S1-AVI & His Recombinant Protein (YP_009724390.1) (Val16–Arg685) were expressed, respectively, with a C-terminally polyhistidine-tagged AVI tag at the C-terminus. The purified proteins were biotinylated in vitro, respectively.

### Conjugation of antigen protein and PE/APC streptavidin oligos

Biotinylated proteins were then conjugated to Biolegend TotalSeq™ PE or APC streptavidin oligos at a 5:1 molar ratio of antigen protein to PE/APC streptavidin oligos. The amount of antigen was chosen based on a fixed amount of 0.6 µg PE or APC streptavidin oligos. The antigen protein and streptavidin oligos were mixed and incubated on ice for 30 min. After incubation, antigen-streptavidin oligos were diluted to a final volume of 10 µL. Antigen-streptavidin oligo diluent was then centrifuged at 14,000 × *g* at 2–8 °C for 10 min. Nine microliter liquid was carefully pipetted out avoiding the bottom of the tube and transferred to a new tube. Antigen-streptavidin oligos were then used immediately for cell staining. 1 × 10^9^ antigen-streptavidin oligos were used in every 1 × 10^6^ cells in 100 µL.

For PBMC, cells were stained with APC anti-human CD3 Antibody (Biolegend, 300312), Brilliant Violet 605™ anti-human CD14 Antibody (Biolegend, 367126), Brilliant Violet 605™ anti-human CD16 Antibody (Biolegend, 302039), FITC anti-human CD19 Antibody (Biolegend, 363008), Brilliant Violet 421™ anti-human CD27 Antibody (Biolegend, 356417), APC/Cyanine7 anti-human IgM Antibody (Biolegend, 314520), Antigen-oligo streptavidin PE cocktail after using Fc Receptor Blocking Solution (Biolegend, 422301). After 30-min incubation on ice, cells were washed twice with PBS plus 2% FBS and stained with eBioscience™ 7-AAD Viability Staining Solution (Invitrogen, 00-6993-50). Stained samples were then collected on an Astrios EQ (BeckMan Coulter) and single CD3^–^, CD14^–^, CD16^–^, 7-AAD^–^, CD19^+^, CD27^+^, antigen^+^ cells were collected. The antigen-specific gates were drawn by use of healthy donor controls. UltraComp eBeads™ Compensation Beads (Invitrogen, 01-2222-41) were used to normalize results.

MACS-selected B cells were stained with Brilliant Violet 605™ anti-human CD14 Antibody (Biolegend, 367126), Brilliant Violet 605™ anti-human CD16 Antibody (Biolegend, 302040), FITC anti-human CD19 Antibody (Biolegend, 392508), Brilliant Violet 421™ anti-human CD27 Antibody (Biolegend, 302824), Antigen-oligo streptavidin APC/PE cocktail after using Fc Receptor Blocking Solution (Biolegend, 422301). After 30-min incubation on ice, cells were washed twice with PBS plus 2% FBS and stained with eBioscience™ 7-AAD Viability Staining Solution (Invitrogen, 00-6993-50). Stained convalescents’ B cell samples were then collected on an Astrios EQ (BeckMan Coulter). Stained CoronaVac vaccinees’ B cell samples were then collected on a FACSAriaIII (BD Biosciences). Single CD14^–^, CD16^–^, 7-AAD^–^, CD19^+^, CD27^+^, antigen^+^ cells were collected. The antigen-specific gates were drawn by use of healthy donor controls. Cells were collected into pre-coated protein-lobind Eppendorf tube containing 500 µL of Ca^2+^/Mg^2+^-free PBS plus 30% FBS. After cell sorting, the volume was made up to 1400 μL by adding ice-cold Ca^2+^/Mg^2+^-free PBS containing 2% FBS, and then the cells were centrifuged at 400 × *g* for 10 min. 1390 µL of supernatant was removed and cells were resuspended in 30 µL of Ca^2+^/Mg^2+^-free PBS containing 2% FBS. 1–2 µL cells were used for counting, and the remaining cells were used for the following 10× procedure.

Flow cytometry data were analyzed with FlowJo 10.7.1.

### Single-cell 5′-mRNA, VDJ, and feature barcode sequencing

VDJ, 5′-mRNA, and feature barcode libraries were prepared using the 10× Chromium System (10X Genomics, Pleasanton, CA) according to the manufacturer’s instructions. The C Chromium Next GEM Single Cell 5′ Kit v2 (10X Genomics, PN-1000263), Chromium Single Cell Human BCR Amplification Kit (10X Genomics, PN-1000253), and 5′ Feature Barcode Kit (10X Genomics, PN-1000256), Library Construction Kit (10X Genomics, PN-1000190), and Dual Index Kit TT Set A (10X Genomics, PN-1000215), Dual Index Kit TN Set A (10X Genomics, PN-1000250) were used. All libraries were quantified by using Qubit 3.0 (Thermo Fisher), Fragment Analyzer, and qPCR. Sequencing was performed on a Hiseq 2500 platform running Rapid SBS Kit V2 2x100bp kit (Illumina, FC-402–4021), with 10 cycles for the i7 index and i5 index. Average sequencing depth aimed for the mRNA library is 20,000 read pairs per cell, 5000 read pairs per cell for the VDJ libraries, and 5000 read pairs for feature barcode libraries.

### In vitro expression of monoclonal antibodies (mAbs)

Representative antibodies from each subject were chosen for synthesis by choosing clonally-enriched cells. Immunoglobulin heavy and light chain genes were obtained by 10× Genomics VDJ sequencing analysis and recombinant mAbs were synthesized by Sino Biologicals Inc. Briefly, sequences were cloned into human IgG1 expression vectors using Gibson assembly, and heavy and light genes were co-transfected into HEK293 cells. Cells were cultured for a duration of 7 days and secreted mAbs were then purified from the supernatant using protein A affinity chromatography.

### Construction of the pseudoviruses

The backbone of the pseudovirus was provided by the VSV G pseudotyped, in which the G gene is replaced with the firefly luciferase (Fluc) reporter gene, and the SARS-CoV-2 spike protein is incorporated.

SARS-CoV-2 spike (GenBank: MN908947) were optimized and inserted into the pcDNA 3.1 to obtain the plasmid pcDNA3.1-SARS-CoV-2-spike (pcDNA3.1.S2.). Mutant plasmids were constructed based on pcDNA3.1.S2. using site-directed mutagenesis. PCR was performed using the SARS-CoV-2 Spike D614G plasmid as a template according to the manual of PrimeSTAR (Takara) reagents. The PCR products were digested and transformed into DH5α competent bacteria (Invitrogen, 12034013). The bacteria were seeded on the plates containing corresponding drugs for resistance selection. After overnight incubation (14–15 h) at 37 °C, single colony of the bacteria was selected and sequenced.

HEK293T cells were digested and adjusted to a concentration of 5–7 × 10^5^ cells/mL. 15 mL cells were transferred in to a T75 cell flask and incubated overnight at 37 °C, 5% CO_2_. When the cell density reached 70%–90% confluence, the culture medium was discarded and 15 mL G*∆G-VSV virus (VSV G pseudotyped virus, Kerafast) of 7.0 × 10^4^ TCID50/mL was used for infection. At the same time, 30 μg of the S protein expression plasmid was transfected according to the instructions of Lipofectamine 3000 (Invitrogen, L3000015), and then the cells were cultured in an incubator at 37 °C and 5% CO_2_ for 6–8 h. After incubation, the culture medium was aspirated, and the cells were gently washed twice with PBS + 1% FBS. Next, 15 mL fresh complete DMEM was added to the flask, and the cells were incubated at 37 °C with 5% CO_2_ for 24 h. Cell supernatant was collected and 15 mL of fresh complete DMEM were added into the 293T cells, which were incubated for another 24 h. Cell supernatant was harvested and mixed with the previous supernatant. After that, the culture supernatant was centrifuged at 1000 × *g* for 10 min, filtered with 0.45-μm filter, aliquoted, and frozen at −80 °C for further use.

### Pseudovirus neutralization assay

The pseudovirus neutralization assays were performed using Huh-7 cell lines (Japanese Collection of Research Bioresources, JCRB0403).^28^ Cells was cultured in DMEM, high glucose (Hyclone, SH30243.01) supplemented with 100 U/mL of Penicillin–Streptomycin solution (Gibco, 15140122), 20 mM HEPES (Gibco, 15630080) and 10% FBS (Gibco, 10099141C) in a 5% CO_2_ environment at 37 °C. Prior to experiments, aliquots of plasma or serum samples were heat-inactivated (56 °C for 30 min). The test samples (plasma/sera/antibodies) were serially diluted using complete DMEM culture media in a 96-well plate for a total of six gradients, and then the virus solution with 1.3 × 10^4^ (1–2 × 10^4^) TCID50/mL was added. Six virus control wells were used. After 1-h incubation at 37 °C and 5% CO_2_, the 96-well plates were seeded with 100 μL of trypsin-treated (0.25% Trypsin-EDTA, Gibco, 25200072) Huh-7 cell (2 × 10^5^ cells/mL). After 24-h incubation at 37 °C and 5% CO_2_, the culture supernatant was aspirated gently to leave 100 μL in each well; then, 100 μL of luciferase substrate (PerkinElmer, 6066769) was added to each well. 150 μL of lysate was mixed and transferred to a white opaque 96-well microplate (PerkinElmer, Ensight, 6005290) for the detection of luminescence using a microplate luminometer (PerkinElmer, Ensight), after incubation at room temperature for 2 min. Each group of plasma/serum samples contained two replicates. IC_50_ were determined by a four-parameter non-linear regression using GraphPad Prism 8.0 (GraphPad Software Inc.).

### Authentic SARS-CoV-2 neutralization CPE assay

A neutralization assay of authentic SARS-CoV-2 (WT: BetaCoV/Beijing/IME-BJ01/2020, GWHACAX01000000, 501Y.V2: CSTR:16698.06.NPRC 2.062100001) was performed using a CPE assay. The CPE assays were performed using Vero cell line (ATCC, CCL-81). Cells was cultured in DMEM, high glucose (Hyclone, SH30243.01) supplemented with 100 U/mL of Penicillin–Streptomycin solution (Gibco, 15140122), 10 mM HEPES (Gibco, 15630080) and 10% FBS (Gibco, 10099141C) in a 5% CO_2_ environment at 37 °C. Prior to experiments, aliquots of plasma or serum samples were heat-inactivated (56 °C for 30 min). The test samples were serially diluted using complete DMEM and incubated with 50 μL of 100 CCID50 authentic SARS-CoV-2 for 1 h at 37 °C. After incubation, the 96-well plates were seeded with 100 μL Vero cells adjusted to a concentration of 1.5 × 10^5^ cells/mL. After incubation of the cells with the mixture for 72 h at 37 °C and 5% CO_2_, CPEs were scored. The 50% neutralization titer was calculated by the Spearman-Karber method. All experiments were performed in a Biosafety Level 3 facility.

### ELISA quantification of mAbs

The purified mAbs were tested for SARS-CoV-2 RBD/spike reactivity by ELISA. ELISA plates were coated with SARS-CoV-2 RBD or S protein at 0.03 μg/mL and 1 μg/mL overnight at 4 °C. Following standard washing and blocking, 100 μL of 1 μg/mL antibodies was added to each well. After a 2-h incubation at room temperature, plates were washed and incubated with 0.08 μg/mL goat anti-human IgG (H + L)/HRP (Jackson Laboratory, 109-036-088) for 1 h at room temperature. Absorbance at 450 nm was measured by a microplate reader. An mAb is defined as ELISA positive when OD450 is saturated using 1 μg/mL RBD/S protein.

### ELISA for plasma/sera

Plasma IgG responses to SARS-CoV-2 S (SARS-CoV-2 Spike S1 + S2 ECD Antibody Titer Assay Kit, Sino Biological Inc., KIT 004), RBD (SARS-CoV-2 Spike RBD Antibody Titer Assay Kit, Sino Biological Inc., KIT 002), NTD (SARS-CoV-2 Spike S1 NTD-His & AVI Recombinant Protein, Sino Biological Inc., 0591-V49H), S1-D614G (SARS-CoV-2 Spike S1(D614G)-His Recombinant Protein, Sino Biological Inc., 40591-V08H3), S1-RBD.V2 (SARS-CoV-2 Spike S1(K417N, E484K, N501Y, D614G)-His Recombinant Protein, Sino Biological Inc., 40591-V08H10), S1–501Y.V2 NTD (SARS-CoV-2 Spike S1(242–244Δ, D614G)-His, Sino Biological Inc., BD61–2) were measured by ELISA. SARS-CoV-2 spike or RBD or NTD or mutant S1 proteins were coated at 1 μg/mL onto 96-well ELISA plates, and incubated overnight at 4 °C. After standard washing and blocking, plasma samples were added at 1:100 and serially diluted by 2-fold in blocking buffer. Following a 2-h incubation at 37 °C, a mouse anti-human IgG horseradish peroxidase-conjugated antibody was added and incubated for 1 h at room temperature. The signal was developed with TMB substrate (Sino Biological Inc., SEKCR-B) for 20 min at room temperature, followed by the standard stop process. Absorbance at 450 nm was measured and OD-blank was used to calculate AUC using Graphpad Prism.

### Protein expression and purification for structural analyses

Sf21 and Hi5 insect cells were maintained at 27 °C and 110 rpm in SIM SF and SIM HF media (Sino Biological), respectively, supplemented with Penicillin–Streptomycin (Gibco). HEK293F cells were maintained at 37 °C and 5% CO_2_ in SMM 293-TI medium (Sino Biological) supplemented with Penicillin–Streptomycin. Protein expression and purification were carried out essentially as previously described.^19^ The RBD of the S protein (residues 319–541) with an N-terminal His6 tag was cloned into a customized pFastBac vector that encodes a gp67 signal peptide. The NTD (residues 13–303) with a C-terminal His6 tag was cloned into a pFastBac vector that encodes a melittin signal peptide. Bacmids were generated using the Bac-to-Bac system. Baculoviruses were generated by transfecting purified bacmids into the Sf21 cells using the X-tremeGENE 9 DNA transfection reagent (Roche) and subsequently amplified using the Sf21 cells. For protein production, Hi5 cells at 1.5–2.0 × 10^6^ cells/mL were infected with the baculoviruses. Culture supernatants were harvested at 48 h post infection, concentrated by a 10 kDa MW cutoff Hydrosart Ultrafilter (Sartorius), and buffer exchanged into 25 mM Tris-HCl, pH 8.0, and 200 mM NaCl. Proteins were purified using the Ni-NTA and size exclusion chromatography. The Fabs BD-508, BD-515, BD-623, N12-9, and N12-11 were obtained by transient expression in the HEK293F cells using polyethylenimine (PEI, Polysciences). Culture supernatants were harvested at 96 h post transfection, and proteins were purified from the conditioned media using the Ni-NTA and size exclusion chromatography.

### Crystallization and structure determination

Crystallization was performed at 18 °C using the sitting-drop vapor diffusion method (Supplementary information, Table S4). Diffraction-quality crystals were obtained in the following conditions:

BD-508/RBD: 0.1 M sodium citrate, pH 5.0, and 11% (w/v) polyethylene glycol 6000.

BD-515/RBD: 0.3 M ammonium sulfate, 0.1 M potassium sodium tartrate tetrahydrate, and 25% (w/v) polyethylene glycol 4000.

BD-623/RBD: 0.2 M ammonium sulfate, and 18% (w/v) polyethylene glycol 8000.

N12-11/NTD: 0.2 M magnesium chloride, 0.1 M Tris-HCl, pH 7.0, and 10% (w/v) polyethylene glycol 8000.

For data collection, the crystals were transferred to a solution containing the crystallization solution supplemented with 10%–20% ethylene glycol, and then flash-cooled in liquid nitrogen. Diffraction data were collected at the Shanghai Synchrotron Radiation Facility (BL17U, BL19U1) and the Photon Factory in Japan (beamline BL-1A). Diffraction data were processed using HKL2000 (HKL Research). Structures were solved by molecular replacement using PHASER^29^ in PHENIX.^30^ Iterative model building and refinement were carried out in COOT^31^ and PHENIX.

### Cryo-EM data collection, processing, and structure building

To prepare the sample for cryo-EM study, 4 μL S6P complexed with the N9 Fab (0.7 mg/mL) and 4 μL BD-368-2/BD-604 Fabs (both at 1.0 mg/mL) were mixed, and then immediately applied onto the glow-discharged holy-carbon gold grids (Quantifoil, R1.2/1.3) using an FEI Vitrobot IV. The grids were flash-cooled in liquid ethane and screened using a 200 kV Talos Arctica. The grids were then transferred to a Titan Krios operated at 300 kV for data collection. Movies were recorded on a K2 Summit direct electron detector (Gatan) using the SerialEM software.^32^ A total of 4442 movies were recorded for image processing. MotionCor2^33^ was used to align and average raw movie frames into motion-corrected images. The CTFFIND4^34^ was used to estimate the contrast transfer function (CTF) parameters of each motion-corrected image. The best 3957 micrographs were manually selected and processed by RELION-3.1.^35^ The cryo-EM map of BD-368-2 Fab in complex with S6P (EMDB ID: EMD-30374) was used as a reference for the 3D classification (Supplementary information, Table S5).

For model building, the NTD structure (PDB: 7CHH) and the BD-604/RBD/BD-368-2 complex structure (PDB: 7CHF) were first docked into the cryo-EM density map using UCSF Chimera.^36^ The structural models were then manually built in Coot and refined using the real-space refinement in PHENIX. Figures were prepared using Pymol (Schrödinger) and UCSF Chimera.

### Single-cell sequencing data analysis

The raw fastq files of single-cell gene expression, feature barcode, and VDJ sequencing were processed using the multi pipeline of 10X Genomics’ Cell Ranger (5.0.0) software with GRCh38 reference genome (Cell Ranger GRCh38 Reference — 5.0.0). The output filtered feature-barcode matrices were input to R software (v3.6.2) with Seurat package (v3.2.0) for downstream analysis.^37^ Cells with < 300 or > 9000 genes, < 500 or > 50,000 UMIs, or > 10% mitochondrial UMIs were filtered out. Cell types were identified using SingleR package with “MonacoImmuneData” as reference.^38,39^

## Supplementary information

Supplementary information, Table S1

Supplementary information, Table S2

Supplementary information, Table S3

Supplementary information, Table S4

Supplementary information, Table S5

Supplementary information, Fig. S1

Supplementary information, Fig. S2

Supplementary information, Fig. S3
