# Supplementary material for: Humoral immune response to circulating SARS-CoV-2 variants elicited by inactivated and RBD-subunit vaccines
Source: Cell Res. 2021 May 21;31(7):732–41. doi: 10.1038/s41422-021-00514-9 (PMC8138844; doi:10.1038/s41422-021-00514-9)
Supplement: Supplementary file 4 — Supplementary information, Table S4 [file 41422_2021_514_MOESM4_ESM.pdf]

Table S4. Data collection and refinement statistics for crystal structures.

	BD-508/RBD (PDB ID: 7E86)	BD-515/RBD (PDB ID: 7E88)	BD-623/RBD (PDB ID: 7E7Y)	N12-11/NTD (PDB ID: 7E7X)
**Data collection**				
Resolution (Å)	2.90	3.15	2.41	2.78
Space group	$C222_1$	$P1$	$P2_12_12_1$	$P2_12_12$
Cell dimensions				
a, b, c (Å)	82.4, 148.7, 147.8	103.7, 107.6, 108.8	93.8, 126.6, 138.9	91.4, 113.3, 210.9
α, β, γ (°)	90.0, 90.0, 90.0	63.3, 81.0, 66.8	90.0, 90.0, 90.0	90.0, 90.0, 90.0
Multiplicity	13.3 (13.4)	3.5 (3.5)	13.5 (13.8)	13.1 (13.2)
Completeness (%)	99.7 (99.4)	96.5 (87.8)	98.1(93.6)	98.7(92.7)
I / σI	9.4 (2.2)	10.0 (2.0)	11.0 (2.1)	15.2 (2.4)
Wilson B-factor	53.8	81.4	38.2	53.3
R_{merge}	0.29 (1.13)	0.07 (0.40)	0.13 (0.74)	0.16 (0.90)
R_{meas}	0.30 (1.18)	0.09 (0.47)	0.14 (0.77)	0.17 (0.94)
R_{pim}	0.08 (0.32)	0.04 (0.24)	0.04 (0.21)	0.04 (0.25)
$CC_{1/2}$	0.98 (0.77)	0.99 (0.90)	0.99 (0.90)	0.99 (0.93)
**Refinement**				
Reflections used in refinement	20474 (2022)	65003 (5893)	63148 (5923)	55215 (5905)
Reflections used for R-free	1980 (190)	2012 (198)	1977 (185)	1988 (183)
R_{work} / R_{free}	0.196/0.255	0.217/0.255	0.198/0.242	0.239/0.278
Number of non-hydrogen atoms	4698	18866	9916	9767
macromolecules	4698	18866	9339	9767
ligand/ion	0	0	0	0
solvent	0	0	577	0
Protein residues	613	2447	1235	1280
RMS(bonds)	0.01	0.011	0.009	0.003
RMS(angles)	1.32	1.44	1.03	0.89
Ramachandran favored (%)	93.53	93.31	96.05	94.86
Ramachandran allowed (%)	6.47	6.69	3.95	5.14
Ramachandran outliers (%)	0	0	0	0
Average B-factor	45.99	89.42	39.04	60.67

Each dataset was collected from a single crystal. Values in parentheses are for highest-resolution shell.
