# Supplementary material for: Humoral immune response to circulating SARS-CoV-2 variants elicited by inactivated and RBD-subunit vaccines
Source: Cell Res. 2021 May 21;31(7):732–41. doi: 10.1038/s41422-021-00514-9 (PMC8138844; doi:10.1038/s41422-021-00514-9)
Supplement: Supplementary file 5 — Supplementary information, Table S5 [file 41422_2021_514_MOESM5_ESM.pdf]

Table S5. Cryo-EM data collection, processing, and validation statistics

	S6P in complex with the Fabs of BD-368-2, BD-604, and N12-9 (PDB:7E8C; EMDB: EMD-31014)	The local structure of NTD-RBD together with the Fabs of BD-368-2, BD-604, and N12-9 (PDB: 7E8F; EMDB: EMD-31017)
**Data collection and processing**		
Voltage (kV)	300	
Microscope	FEI Titan Krios G3	
Camera	K2 Summit (Gatan)	
Magnification (calibrated)	130,000X	
Electron exposure ($e^{-}/Å^2$)	63.27	
Exposure rate ($e^{-}/Å^2/s$)	7.6046	
Number of frames collected per micrograph	32	
Energy filter slit width	20 eV	
Automation software	SerialEM	
Defocus range (μm)	–0.6 to –1.2	
Pixel size (Å)	1.052	
Micrographs used	3,957	
Symmetry imposed	C1	C1
Initial particle images	858,315	535,671
Final particle images	178,557	304,696
Resolution at 0.5 FSC of masked reconstruction (Å)	3.79	3.66
Resolution at 0.143 FSC of masked reconstruction (Å)	3.16	3.18
Map sharpening B factor ($Å^2$)	–70.48	–113.22
**Refinement**		
Initial model used (PDB code)	7CHH, 7CHF	7CHH, 7CHF
Refinement package	Phenix v1.18 (Real-space refinement at 3.16 Å)	Phenix v1.18 (Real-space refinement at 3.18 Å)
Map-model CC		
CC_mask	0.70	0.81
CC_box	0.71	0.68
CC_peaks	0.57	0.62
CC_volume	0.69	0.79
Model composition		
Non-hydrogen atoms	40,225	8,768
Protein residues	5,218	1,134
Ligands	0	0
R.m.s. deviations		
Bond lengths (Å)	0.009	0.005
Bond angles (°)	0.872	0.674
B factors ($Å^2$)		
Protein	73.71	64.92
Validation		
MolProbity score	2.71	1.95
Clashscore	14.31	8.89
Poor rotamers (%)	6.48	0

Ramachandran plot		
Favored (%)	93.37	92.45
Allowed (%)	6.61	5.24
Disallowed (%)	0.02	0
Cβ outliers (%)	0.00	0.00
CaBLAM outliers (%)	3.34	3.21
