# Supplementary material for: Humoral immune response to circulating SARS-CoV-2 variants elicited by inactivated and RBD-subunit vaccines
Source: Cell Res. 2021 May 21;31(7):732–41. doi: 10.1038/s41422-021-00514-9 (PMC8138844; doi:10.1038/s41422-021-00514-9)
Supplement: Supplementary file 6 — Supplementary information, Fig. S1 [file 41422_2021_514_MOESM6_ESM.pdf]

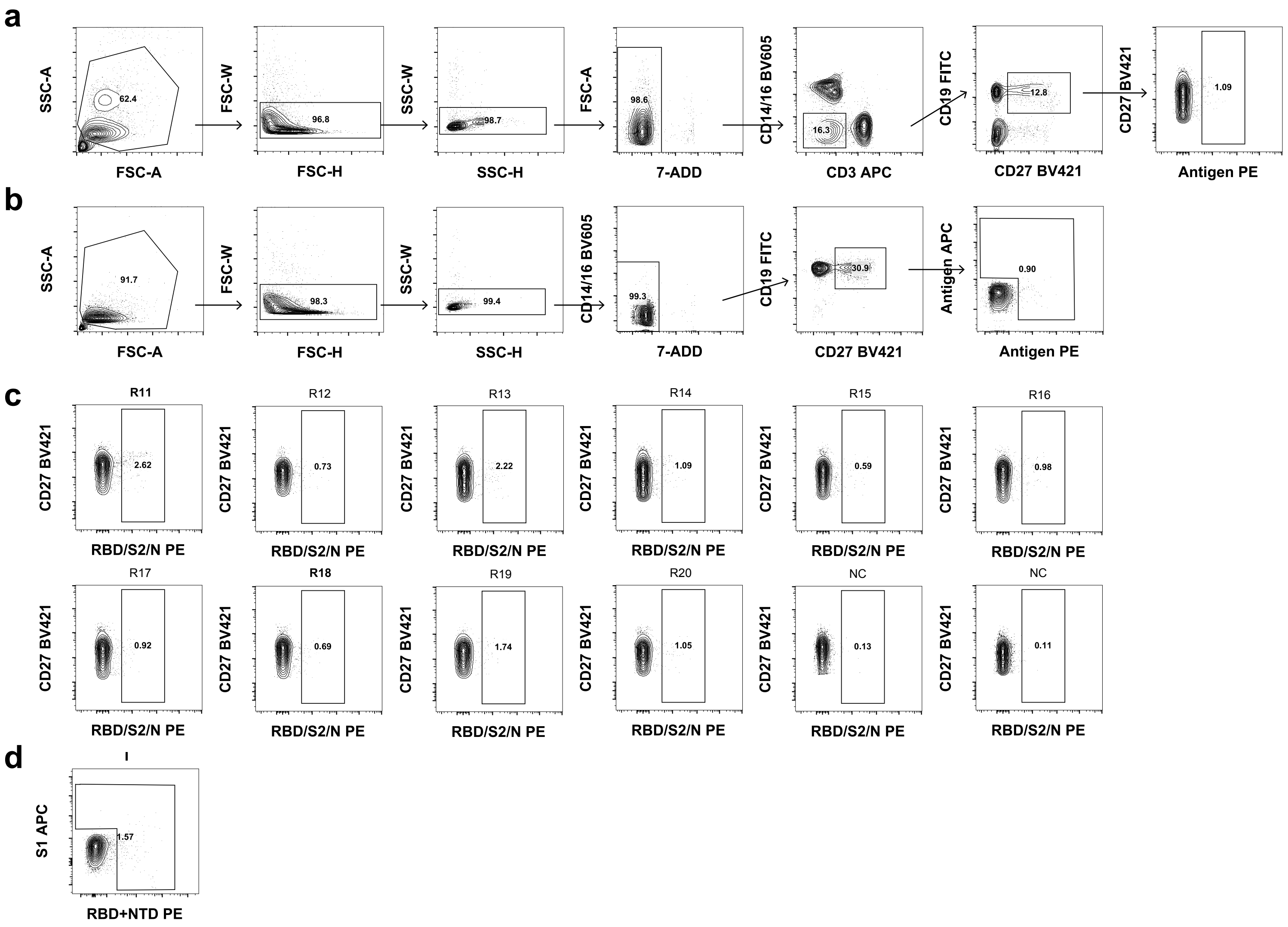

a
SSC-A
FSC-A
62.4
FSC-W
FSC-H
96.8
SSC-W
SSC-H
98.7
FSC-A
7-ADD
98.6
CD14/16 BV605
CD3 APC
16.3
CD19 FITC
CD27 BV421
12.8
CD27 BV421
Antigen PE
1.09
b
SSC-A
FSC-A
91.7
FSC-W
FSC-H
98.3
SSC-W
SSC-H
99.4
CD14/16 BV605
7-ADD
99.3
CD19 FITC
CD27 BV421
30.9
Antigen APC
Antigen PE
0.90
c
R11
2.62
R12
0.73
R13
2.22
R14
1.09
R15
0.59
R16
0.98
R17
0.92
R18
0.69
R19
1.74
R20
1.05
NC
0.13
NC
0.11
CD27 BV421
RBD/S2/N PE
d
I
S1 APC
RBD+NTD PE
1.57

Fig. S1 Flow cytometry sorting of antigen-positive memory B cells.

a. Gating strategy used for PBMC sample. Gating was on singlets that were 7AAD-CD3-CD14-CD16-CD19+CD27+. Sorted cells were antigen positive. **b.** Gating strategy used for MACS sorted B cells sample. Gating was on singlets that were 7AAD-CD14-CD16-CD19+CD27+. Sorted cells were antigen positive. **c.** Examples of cell sorting from ZF2001 vaccinees' PBMC (R11-R20) and healthy donors' PBMC (NC). **d.** Examples of cell sorting from Coronavac vaccinees MACS sorted B cells (I).
