# Supplementary material for: Humoral immune response to circulating SARS-CoV-2 variants elicited by inactivated and RBD-subunit vaccines
Source: Cell Res. 2021 May 21;31(7):732–41. doi: 10.1038/s41422-021-00514-9 (PMC8138844; doi:10.1038/s41422-021-00514-9)
Supplement: Supplementary file 7 — Supplementary information, Fig. S2 [file 41422_2021_514_MOESM7_ESM.pdf]

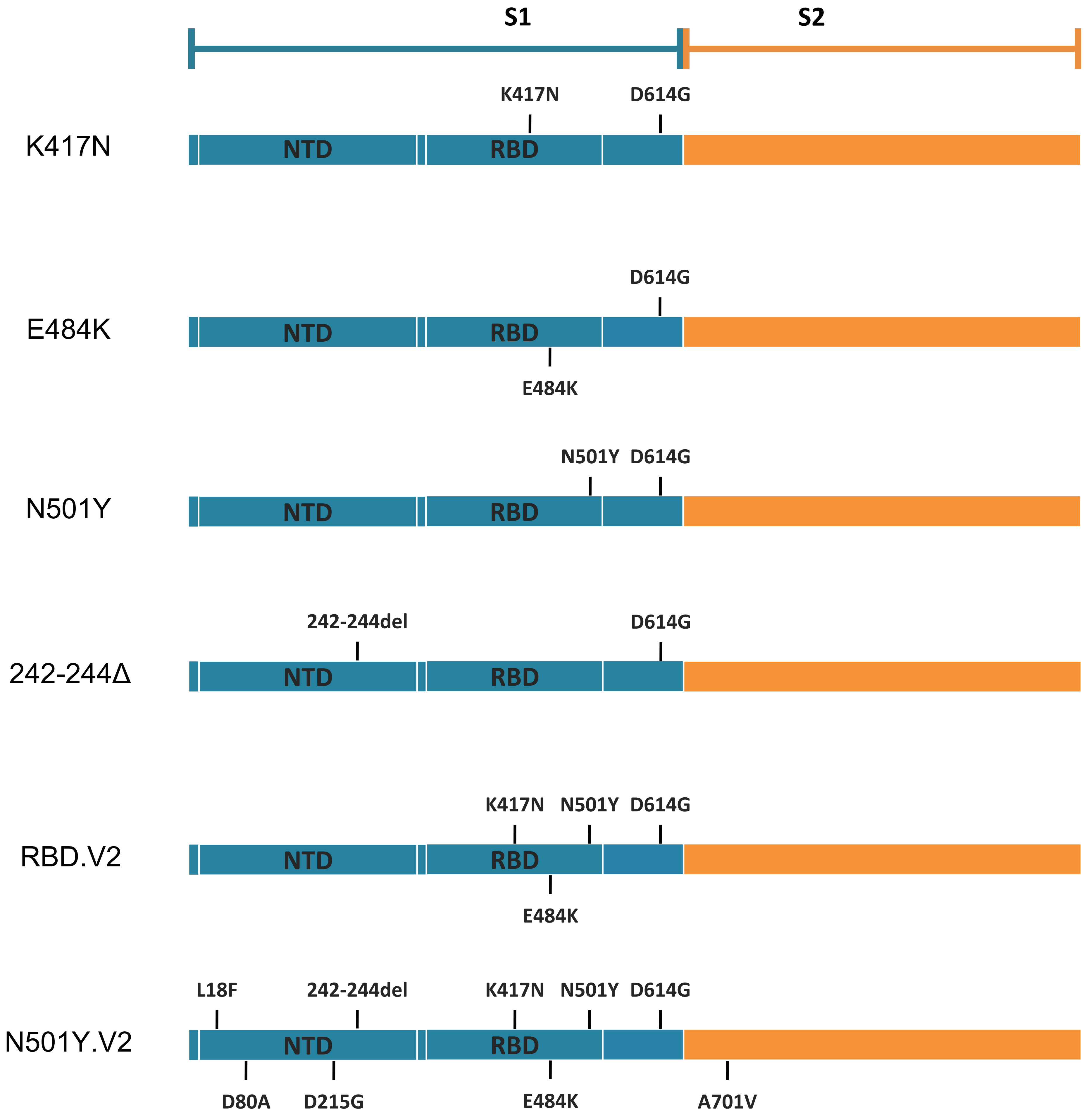

S1
S2
K417N
K417N
D614G
NTD
RBD
E484K
D614G
NTD
RBD
E484K
N501Y
N501Y
D614G
NTD
RBD
242-244Δ
242-244del
D614G
NTD
RBD
RBD.V2
K417N
N501Y
D614G
NTD
RBD
E484K
N501Y.V2
L18F
242-244del
K417N
N501Y
D614G
NTD
RBD
D80A
D215G
E484K
A701V

Fig. S2 Illustration of the pseudoviruses bearing SARS-CoV-2 S mutations.
A total of 6 VSV-pseudovirus carrying RBD and NTD mutants of 501Y.V2 is constructed and used in this study.
