# Supplementary material for: Humoral immune response to circulating SARS-CoV-2 variants elicited by inactivated and RBD-subunit vaccines
Source: Cell Res. 2021 May 21;31(7):732–41. doi: 10.1038/s41422-021-00514-9 (PMC8138844; doi:10.1038/s41422-021-00514-9)
Supplement: Supplementary file 8 — Supplementary information, Fig. S3 [file 41422_2021_514_MOESM8_ESM.pdf]

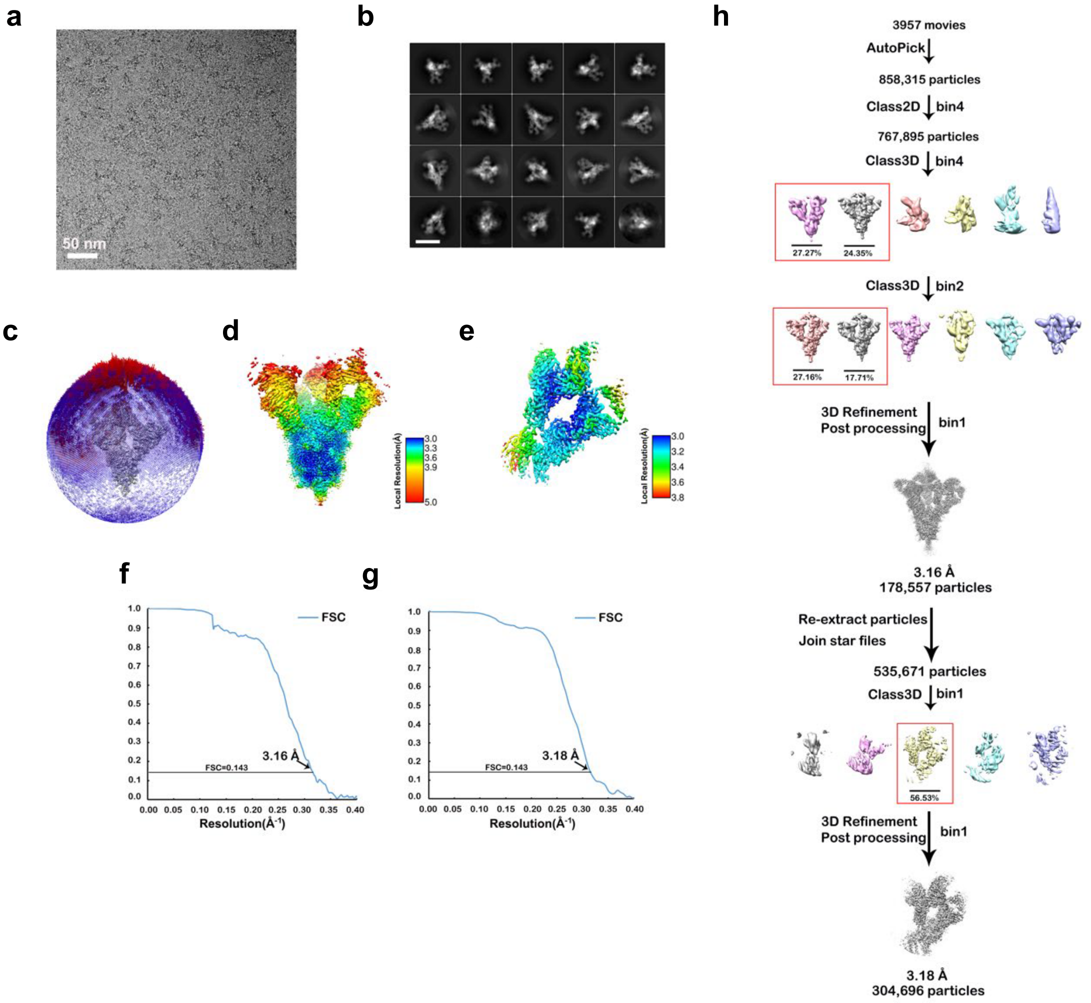

a
50 nm
b
c
d
Local Resolution(Å)
3.0
3.3
3.6
3.9
5.0
e
Local Resolution(Å)
3.0
3.2
3.4
3.6
3.8
f
FSC
3.16 Å
FSC=0.143
Resolution(Å^{-1})
g
FSC
3.18 Å
FSC=0.143
Resolution(Å^{-1})
h
3957 movies
AutoPick
858,315 particles
Class2D bin4
767,895 particles
Class3D bin4
27.27%
24.35%
Class3D bin2
27.16%
17.71%
3D Refinement
Post processing
bin1
3.16 Å
178,557 particles
Re-extract particles
Join star files
535,671 particles
Class3D bin1
56.53%
3D Refinement
Post processing
bin1
3.18 Å
304,696 particles

Fig. S3. Workflow for the 3D reconstruction of the cryo-EM structure of the S6P trimer in complex with the Fabs of BD-368-2, BD-604, and N12-9.

a. A representative raw image collected using a Titan Krios 300 kV equipped with a K2 detector. **b.** Representative 2D classes. **c.** Eulerian angle distribution of the particles used in the final 3D reconstruction. **d.** Local resolution estimation of the overall density map. **e.** Local resolution estimation of the local density map around the region containing the NTD, RBD, and three Fabs. **f.** Gold standard Fourier shell correlation (FSC) curve with the estimated resolution for the overall density map. **g.** FSC curve with the estimated resolution for the local density map. **h.** Flow chart of image processing.
